# Molecular mechanism of radiation tolerance in lung adenocarcinoma cells using single‐cell RNA sequencing

**DOI:** 10.1111/jcmm.18378

**Published:** 2024-05-17

**Authors:** Feiyun Chang, Bozhou Xi, Xinchun Chai, Xiuyan Wang, Manyuan Ma, Yafeng Fan

**Affiliations:** ^1^ Department of Thoracic Surgery, Shanxi Province Cancer Hospital/Shanxi Hospital Affiliated to Cancer Hospital Chinese Academy of Medical Sciences/Cancer Hospital Affiliated to Shanxi Medical University Taiyuan China; ^2^ The Second Clinical Medical School Shanxi Medical University Taiyuan China; ^3^ Shenzhen Engineering Center for Translational Medicine of Precision Cancer Immunodiagnosis and Therapy, Shenzhen YuceBioTechnology Co., Ltd Shenzhen China; ^4^ Department of Respiration, Shanxi Province Cancer Hospital/Shanxi Hospital Affiliated to Cancer Hospital Chinese Academy of Medical Sciences/Cancer Hospital Affiliated to Shanxi Medical University Taiyuan China

**Keywords:** cell subpopulation, lung adenocarcinoma, radiotolerance, single‐cell RNA sequencing, TP53

## Abstract

The efficacy of radiotherapy, a cornerstone in the treatment of lung adenocarcinoma (LUAD), is profoundly undermined by radiotolerance. This resistance not only poses a significant clinical challenge but also compromises patient survival rates. Therefore, it is important to explore this mechanism for the treatment of LUAD. Multiple public databases were used for single‐cell RNA sequencing (scRNA‐seq) data. We filtered, normalized and downscaled scRNA‐seq data based on the Seurat package to obtain different cell subpopulations. Subsequently, the ssGSEA algorithm was used to assess the enrichment scores of the different cell subpopulations, and thus screen the cell subpopulations that are most relevant to radiotherapy tolerance based on the Pearson method. Finally, pseudotime analysis was performed, and a preliminary exploration of gene mutations in different cell subpopulations was performed. We identified HIST1H1D+ A549 and PIF1+ A549 as the cell subpopulations related to radiotolerance. The expression levels of cell cycle‐related genes and pathway enrichment scores of these two cell subpopulations increased gradually with the extension of radiation treatment time. Finally, we found that the proportion of TP53 mutations in patients who had received radiotherapy was significantly higher than that in patients who had not received radiotherapy. We identified two cellular subpopulations associated with radiotherapy tolerance, which may shed light on the molecular mechanisms of radiotherapy tolerance in LUAD and provide new clinical perspectives.

## 1 INTRODUCTION

Lung cancer is a malignant solid tumour with the highest incidence and mortality worldwide, the most important subtype of which is non‐small cell lung cancer (NSCLC), accounting for about 85% of all lung cancer cases.^1^, ^2^ Lung adenocarcinoma (LUAD) is the most common histological subtype of NSCLC. How to effectively diagnose and treat LUAD has always been a difficult problem in the medical field.^3^, ^4^ The prognosis of LUAD is not ideal, and the 5‐year survival rate of most patients usually remains below 15%.^5^ At present, due to the progress of diagnosis and lifestyle changes, the incidence of LUAD has increased slightly, and more than 70% of patients have been diagnosed when they have advanced to the middle and late stages.^6^, ^7^ Radiation therapy is a useful and commonly used therapeutic tool for lung cancer patients, and it plays a key role in achieving local tumour control while limiting damage to surrounding tissues.^8^ Although only a portion of individuals experience positive results from radiation treatment, deciphering the molecular factors that influence how individuals respond to cancer therapy remains a significant obstacle in the field of oncology.

At present, with the progress of medical level, in addition to the conventional therapy of surgical resection of the lesion, chemotherapy and radiotherapy, a variety of targeted drugs and immunotherapy have also appeared a relatively ideal prospect.^9^ As a more traditional and mature treatment method, radiotherapy is widely used in the treatment of LUAD, liver cancer, pancreatic cancer and other cancers.^10^, ^11^, ^12^ Radiation therapy is considered to be the most appropriate treatment for local solid tumours, and in the past half century, radiation therapy has been the mainstream therapy for cancer, and has achieved relatively effective results.^13^ Radiotherapy is also an effective treatment for LUAD when surgery is not possible, and stereotactic radiotherapy has always been the standard treatment for LUAD, and the local curative effect often meets the expectations.^14^ However, radiotolerance is a fundamental problem faced by many kinds of tumour radiotherapy, which may lead to poor curative effect in the later stage. However, the specific mechanism of radiotolerance has not been studied clearly.^15^ This may be related to the radiation activation of stromal cells and tumour stem cells in the tumour microenvironment to produce radiotolerance.^16^ A research investigating molecular predictive markers in sarcoma has identified notable variances in gene patterns among individuals who respond poorly versus those who respond well to radiotherapy.^17^ Ting Zhang et al. found that a circular RNA is involved in the regulation of genes related to pyrodeath of cells to affect the effect of radiotherapy, and knocking out the gene encoding the circular RNA can significantly improve the efficacy of LUAD.^18^

Therefore, it is very important to study the causes and mechanisms of tumour radiotolerance for LUAD radiotherapy. For the first time, we used single‐cell RNA sequencing (scRNA‐seq) technology in order to reveal the potential role of radiation tolerance in LUAD patients. This study will reveal the molecular mechanism of tumour radiotolerance by identifying and studying the subpopulations of cells related to radiotolerance, and help improve the clinical treatment strategy of LUAD.

## 2 MATERIALS AND METHODS

### 2.1 Download of scRNA‐seq data

We download the GSE211617 dataset from the Gene Expression Omnibus (GEO) database,^19^ including purified A549 cells(control) and 6 Gy γ‐ray treated purified A549 cells at 2 h (IR_2h) and 6 h (IR_6h) post‐irradiation. Purified A549 cells were monoclonally cultured in DMEM medium. Irradiated A549 cells for scRNA‐seq were harvested after continued culturing for 2 and 6 h after irradiation. The database was built on 10x Genomics and the sequencing platform was Illumina NovaSeq 6000.

### 2.2 Download of TCGA‐LUAD expression profile and mutant profile

We downloaded RNA‐seq data of TCGA‐LUAD with expression value log2(fpkm+1) from UCSC Xena database (http://xena.ucsc.edu/), and clinical information on whether to receive radiotherapy.^20^ We use the GDCquery function of the TCGAbiolinks package to acquire the single nucleotide variation data of TCGA‐LUAD.^21^ Parameter setting (data. category = ‘Simple Nucleotide Variation’, data. type = ‘Masked Somatic Mutation’, access = ‘open’).

### 2.3 Filtration, standardization, dimensionality reduction and clustering of scRNA‐seq date

We employed an analysis technique utilizing the Seurat package in R for scRNA‐seq data processing, which introduces slight modifications to previously established methods.^22^ This involved the filtration of low‐quality cells through criteria slightly divergent from standard practices, such as the exclusion of cells based on the percentage of mitochondrial gene expression, with cells exceeding a range of 5%–20% being removed. Furthermore, the approach also entailed the elimination of cells exhibiting either an exceptionally high or low number of expressed genes, specifically those with fewer than 200 genes, to maintain data integrity. Subsequently, we normalized the different datasets using SCTransform to eliminate the influence of technical effects between cells and datasets. Principal component analysis (PCA) was used to reduce the number of dimensions of each cell represented (dims = 1:30), and the ‘FindNeighbors’ and ‘FindClusters’ functions with resolutions of 0.1 were used to cluster all cells. Finally, it was visualized with a UMAP diagram.

### 2.4 Differential expression analysis among cell subpopulations

To explore the heterogeneity of gene expression patterns among cell subpopulations, we used the FindAllMarkers function to calculate the highly expressed genes for each cell subset (only.pos = T, min.pct = 0.25, logfc.threshold = 0.25).^23^

### 2.5 The enrichment score of A549 cell subpopulations in TCGA‐LUAD samples was computed

We applied the ssGSEA algorithm from the GSVA package to calculate the enrichment scores of gene sets corresponding to highly expressed genes in A549 cell subpopulations within each TCGA‐LUAD sample. These enrichment scores serve as proxies for the degree of infiltration or representation of A549 subpopulations within the LUAD samples.^24^

### 2.6 The enrichment score of hallmark by TCGA‐LUAD was calculated

We first downloaded 50 hallmark gene sets from MsigDB database, and then calculated the enrichment score of each hallmark gene set by each TCGA‐LUAD sample using the ssGSEA algorithm of GSVA package.^25^

### 2.7 Construction of single‐cell pseudotime trajectories

We used monocle2 package to read the count data of the expression matrix,^26^ combined the phenotype information of the cells, constructed the cds object with the newCellDataSet function and then filtered out the genes expressed in less than 10 cells. We then used the FindAllmarkers function to calculate the differential expression of genes between Ctrl, IR_2h, and IR_6h (*p*_val_adj < 0.05). Next, the reduceDimension function (max_components = 2, method = ‘DDRTree’) was used to reduce the dimension, and the orderCells function was used to order the cells and complete the trajectory construction. Here, we set more branches of cells in the control group as the starting point of the trajectory. Finally, we used differentialGeneTest function (fullModelFormulaStr = ‘~sm.ns(Pseudotime)’) to calculate pseudotime‐dependent genes (*q* < 0.05), the plot_pseudotime_heatmap function was used to draw a heat map of the expression of these genes with pseudotime.

### 2.8 The hallmark enrichment score of each A549 cell was calculated

In order to calculate the hallmark enrichment score of each A549 cell, we first downloaded the h.all.v2023.1.Hs.symbols.gmt file from the MsigDB database and then used the AUCell package to calculate the hallmark enrichment score of each A549 cell.^27^

### 2.9 TCGA‐LUAD mutation spectrum waterfall map

According to the information provided by the official website of TCGA on whether or not they had received radiotherapy, we first divided TCGA‐LUAD into two groups: those who had received radiotherapy and those who had not. The maf file was read using the Read. maf function of the maftools package, and the waterfall map of the top 20 genes with the highest mutation frequency was displayed using the oncoplot function.^28^

### 2.10 Statistical analysis

All calculations were performed in R (version 4.3.1). Notably, we compared the differences in continuous variables between the two groups by using the Wilcoxon rank sum test. The chi‐squared test was used to compare the variability of categorical variables between the two groups. Pearson's method was used to measure the correlation between the continuous variables of the two groups. *p* < 0.05 is considered significant. Sangerbox (http://sangerbox.com/home.html) also offered auxiliary analysis in this paper.

## 3 RESULTS

### 3.1 Single cell atlas of A549 cell line treated with γ rays

**FIGURE 1:**
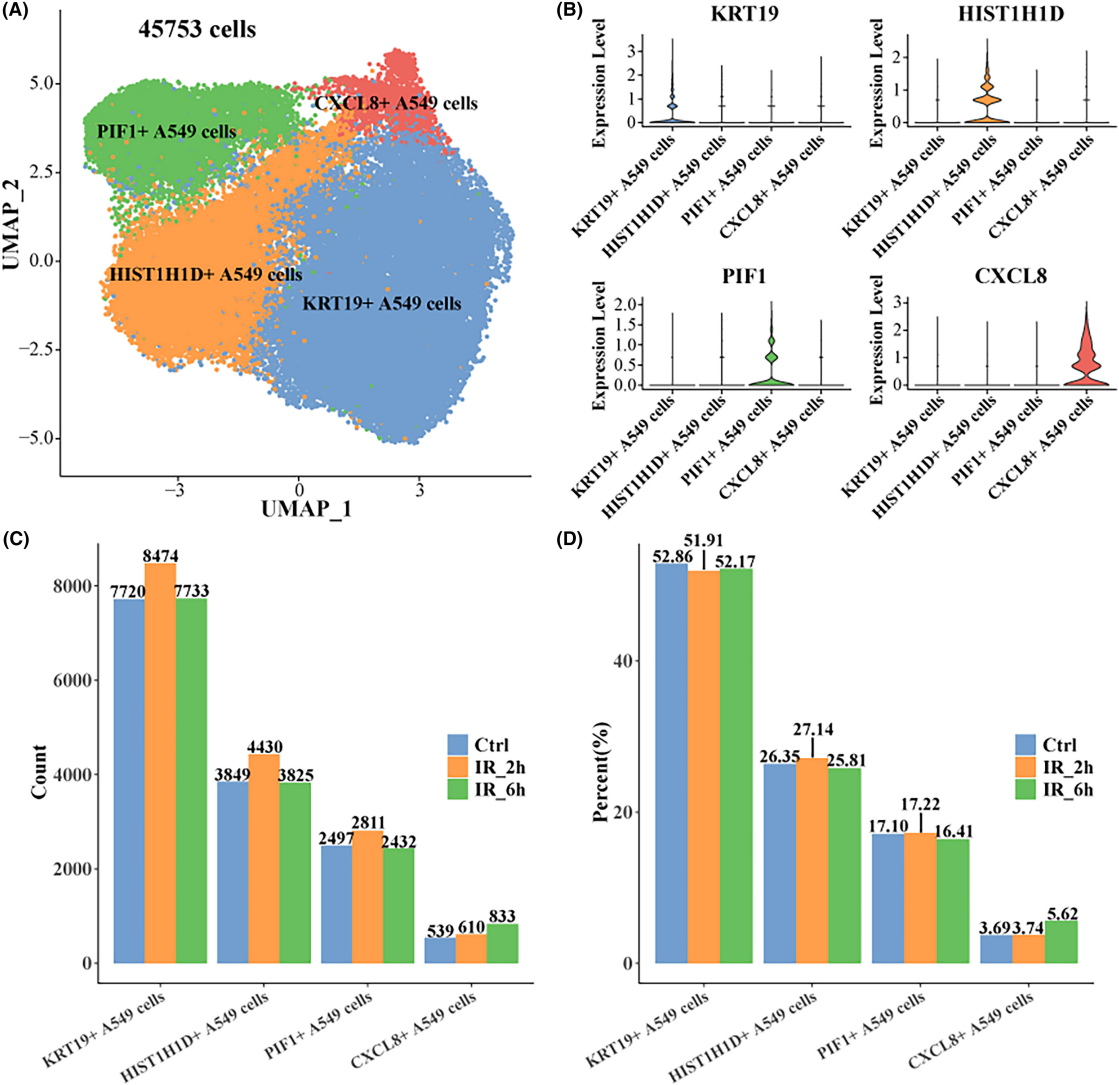
Heterogeneity of A549 cell line after γ‐ray treatment. (A) UMAP map of A549 cell line clustering after γ‐ray treatment. (B) Violin map of specific high‐expression genes of each A549 cell subpopulation. (C) Numbers in control, IR_2h and IR_6h of each A549 cell subpopulation. (D) Proportions in control, IR_2h and IR_6h of each A549 cell subpopulation.

According to the official tutorial of Seurat, after cell filtration, standardization, dimensionality reduction and clustering (Figure S1), 45,753 cells remained and four major cell subpopulations were identified. They were identified as KRT19+ A549 cells, HIST1H1D+ A549 cells, PIF1+ A549 cells and CXCL8+ A549 cells, and the specific high‐expression genes of each subpopulation were analysed (Figure 1A,B). Then we counted the number and proportion of each cell subpopulation treated with radiation for 2 and 6 h, respectively, and found that the number and proportion of CXCL8+ A549 cells treated with radiation for 6 h were slightly higher than those in the other two groups (Figure 1C,D). There was little difference in the proportion of the other three subpopulations within each group, indicating that there was no significant change in the proportion of the cell subpopulations of the A549 cell line over the course of 6 h of γ radiation treatment. These results further confirm the significant heterogeneity of radiotherapy‐treated LUAD cells, which warrants a deeper exploration of their potential role.

### 3.2 Identification of cell subpopulations associated with radiotolerance within TCGA‐LUAD

**FIGURE 2:**
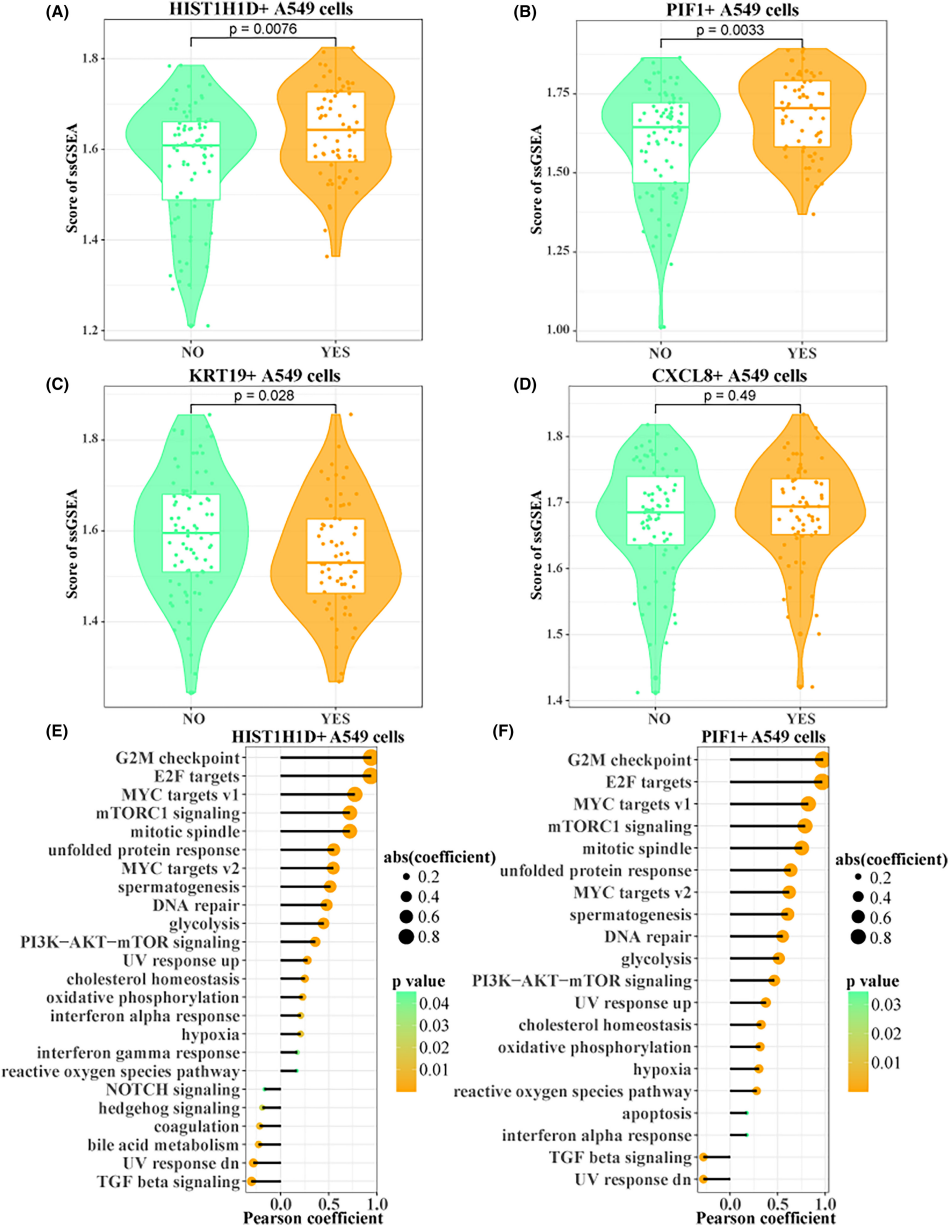
Identification of A549 cell subpopulations associated with radiotolerance in TCGA‐LUAD. (A–D) Box plot of ssGSEA enrichment scores for each A549 cell subpopulation between TCGA‐LUAD with and without radiotherapy. HIST1H1D+ A549 cells (A), PIF1+ A549 cells (B), KRT19+ A549 cells (C), CXCL8+ A549 cells (D). (E, F) Pearson correlation of HIST1H1D+ A549 cells and PIF1+ A549 cells with ssGSEA enrichment scores in hallmark sets.

To further identify the subpopulations of cells associated with radiotolerance, we downloaded the TCGA‐LUAD expression profile and divided patients into those who received radiotherapy and those who did not based on the clinical information provided. Observing the degree of infiltration of A549 cell subpopulations, it was interesting to find that HIST1H1D+ A549 cell and PIF1+ A549 cell enrichment scores in the samples receiving radiotherapy were significantly higher than those in the samples not receiving radiotherapy (Figure 2A,B). In addition, we observed higher enrichment scores in KRT19+ A549 cells in patients who did not receive radiotherapy relative to those who did (Figure 2C). Obviously, in CXCL8+ A549 cells, there was no significant difference between the two groups (Figure 2D). Therefore, to further explore the mechanisms affecting radiotherapy tolerance in LUAD patients, we only initially selected HIST1H1D+ A549 cells and PIF1+ A549 cells for further exploration. In order to further explore the molecular mechanism of radiotolerance of these two cell subpopulations, we calculated the Pearson correlation between HIST1H1D+ A549 cells and PIF1+ A549 cells and ssGSEA enrichment score of hallmark set, respectively. Both cell subpopulations were found to be highly positively correlated with G2M checkpoint, E2F targets, MYC targets v1 and mTORC1 signalling (Figure 2E,F). These results suggest that tumour cells receiving radiation therapy may be able to tolerate radiation therapy by enhancing the activity of mitosis‐related pathways.

### 3.3 Differentiation trajectories of cell subpopulations associated with radiotolerance

**FIGURE 3:**
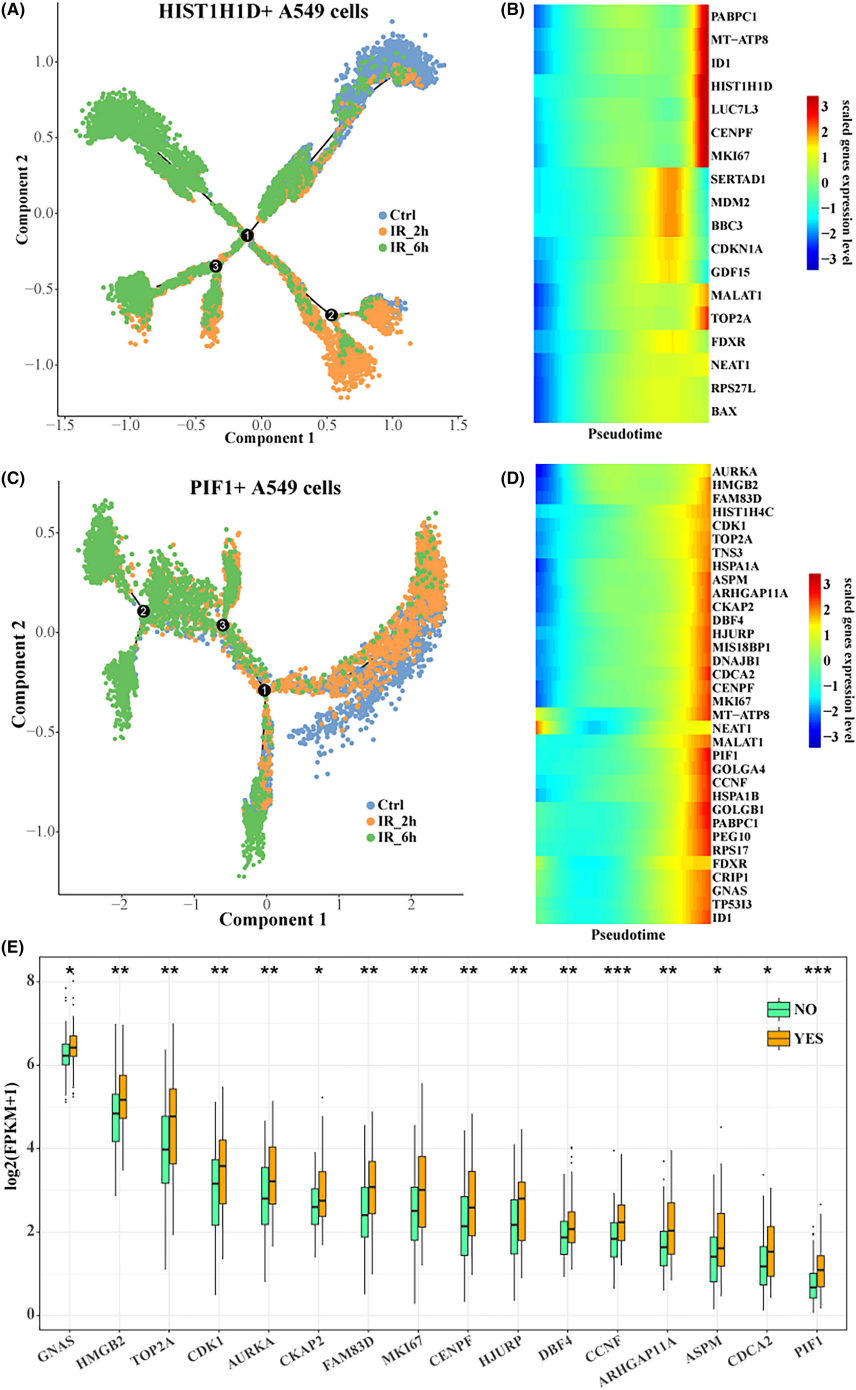
Differentiation trajectories of radiotolerance‐related cell subpopulations. Differentiation trajectory of (A) HIST1H1D+ A549 and (C) PIF1+ A549 cells with radiation time. Heat map of gene expression in (B) HIST1H1D+ A549 and (D) PIF1+ A549 cells with pseudotime. (E) Box map of pesudotime‐related gene expression between radiotherapy and no radiotherapy in TCGA‐LUAD.

In order to further characterize the molecular mechanism of differentiation and evolution of HIST1H1D+ A549 cells and PIF1+ A549 cells during 6 h of γ‐ray treatment, we constructed the differentiation trajectories of HIST1H1D+ A549 cells and PIF1+ A549 cells, respectively. Here, we used the more numerous end of the control cell as the starting point of the trajectories (Figure 3A,C). We found that the expression levels of many cell cycle‐related genes in the two subpopulations gradually increased with pseudotime, such as PABPC1, MT‐ATP8, ID1, HIST1H1D, CENPF and MKI67 (Figure 3B,D). Furthermore, the expression of these genes was also higher in patients who had received radiotherapy in TCGA‐LUAD than in patients who had not received radiotherapy (Figure 3E). These cell cycle‐related genes are strongly associated with radiotolerance.

### 3.4 Radiotolerant cell subpopulations associated with pseudotime hallmark

**FIGURE 4:**
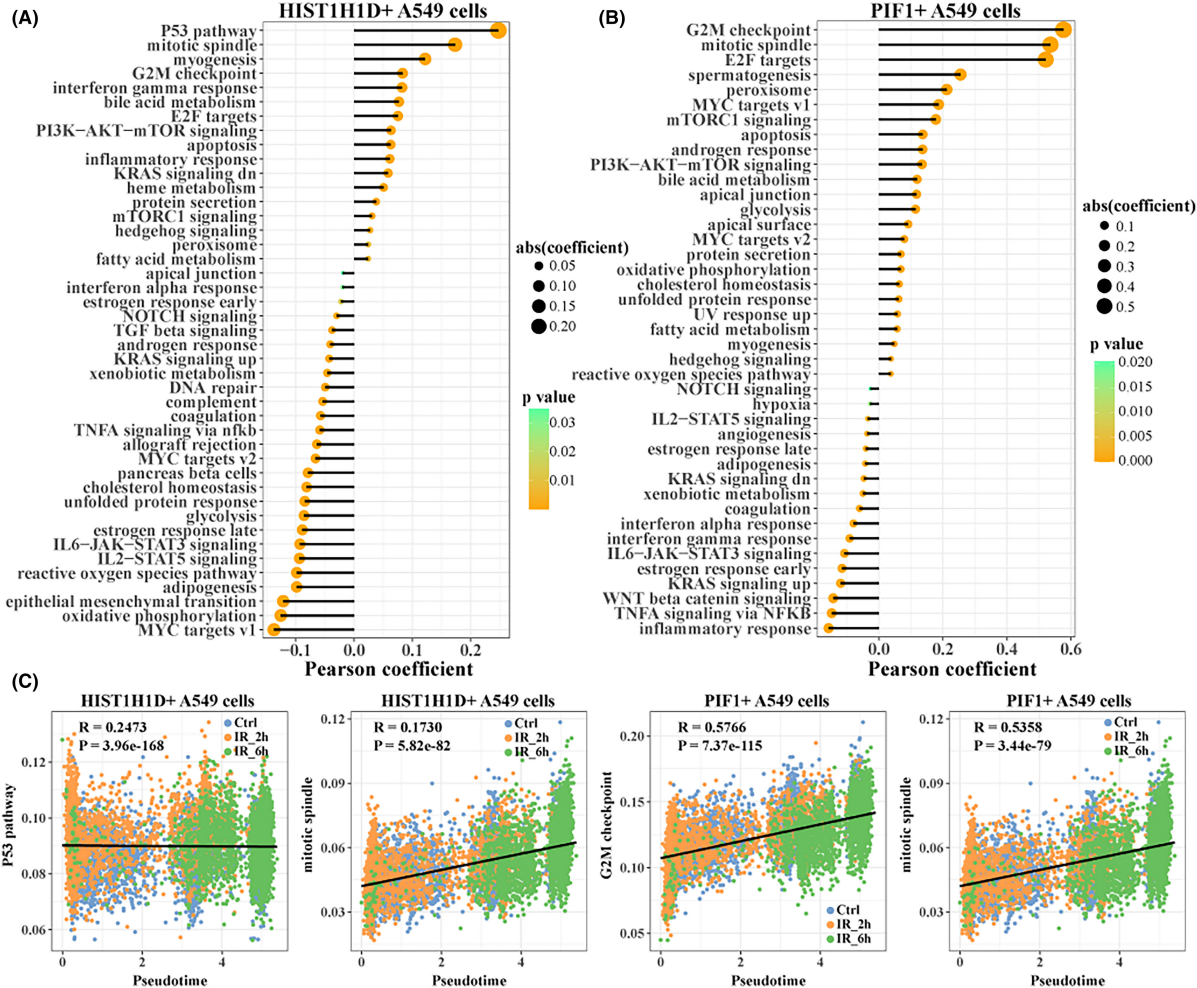
Pseudotime‐associated hallmark within radiotolerance‐related cell subpopulations. Pearson correlation of ssGSEA enrichment scores of pseudotime and hallmark in (A) HIST1H1D+ A549 and (B) PIF1+ A549 cells. (C) HIST1H1D+ A549 cell and PIF1+ A549 cell correlation scatterplot between ssGSEA enrichment scores of cell cycle‐associated hallmark and pseudotime.

In order to further explore the changes of hallmark activity in the two cell subpopulations with the extension of radiation treatment time, we calculated the Pearson correlation between ssGSEA enrichment score of hallmark and pseudotime in HIST1H1D+ A549 cells (Figure 4A) and PIF1+ A549 cells (Figure 4B). Interestingly, in HIST1H1D+ A549 cells and PIF1+ A549 cells, there were significant positive correlations between pseudotime and P53 pathway, mitotic spindle, cell cycle‐related pathways such as G2M checkpoint (Figure 4C). The results indicated that the activity of cell cycle‐related pathways in HIST1H1D+ A549 cells and PIF1+ A549 cells was gradually enhanced with the development of radiation treatment.

### 3.5 TCGA‐LUAD radiotolerance is associated with TP53 mutation

**FIGURE 5:**
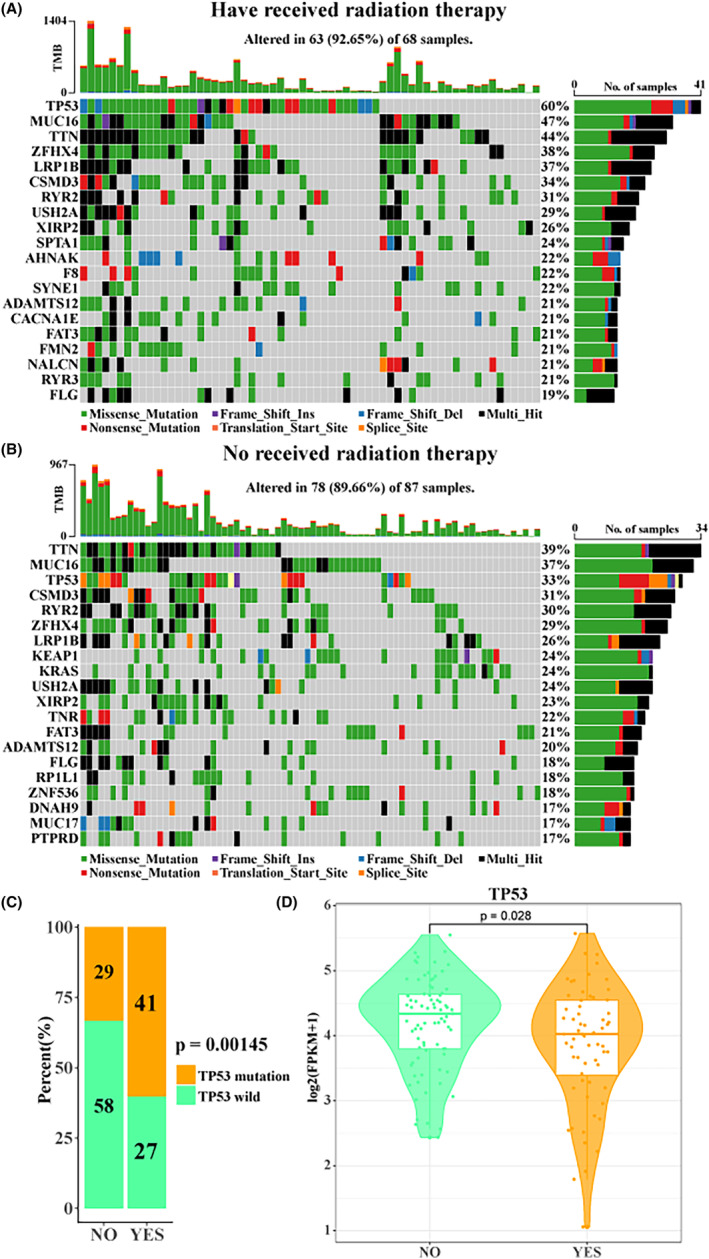
TCGA‐LUAD radiotolerance is associated with TP53 mutation. Waterfall map of the top 20 genes with mutation frequency (A) in patients who received radiotherapy in TCGA‐LUAD and (B) in patients who did not receive radiotherapy in TCGA‐LUAD. (C) Proportional histogram of whether TP53 mutations occurred in patients who received and did not receive radiotherapy in TCGA‐LUAD. (D) Box plot of TP53 expression levels between patients who received and did not receive radiotherapy in TCGA‐LUAD.

Finally, in order to further explore the correlation between radiotolerance and gene mutations, we showed the landscape of the top 20 genes with the highest mutation frequency in patients who received radiotherapy and those who did not through TCGA‐LUAD mutation data (Figure 5A,B). Interestingly, we found that TP53 had the highest mutation frequency in patients who had received radiation therapy (60%), but only 33% in patients who had not received radiation therapy, and a majority of mutations are missense‐type at ‘hot spots’. In particular, we found that the proportion of TP53 mutations in patients who had received radiotherapy was significantly higher than those who had not (*p* = 0.00145, Figure 5C). These results indicated that TP53 mutation was strongly correlated with radiotolerance. In particular, we observed a significant correlation between radiotherapy and reduced TP53 expression levels in our patient cohort (*p* = 0.028, Figure 5D). Although TP53 is a tumour suppressor gene, the lower levels of TP53 expression observed in LUAD patients after radiotherapy do not directly indicate mutations. Therefore, further studies are needed in the future to determine whether these reduced expression levels are the result of TP53 mutations or other regulatory mechanisms affected by radiotherapy. In addition, the activity and gene expression levels of many cell cycle‐related pathways increased with the extension of radiation treatment time as a result of the above differentiation and evolution.

## 4 DISCUSSION

Radiotherapy, a common and beneficial therapeutic technique, is utilized to achieve local tumour control without causing harm to nearby tissues. It is a key component in the management of lung cancer.^8^ However, lower radiotherapy tolerance can severely limit the effectiveness of radiotherapy. To this end, our study utilizes, for the first time, single‐cell RNA sequencing and characterizes in detail the gene expression profiles of cellular subpopulations associated with radiotolerance. In this study, we found that two subpopulations (HIST1H1D+ A549 cells and PIF1+ A549 cells) were significantly correlated with radiotolerance. Enrichment results showed that both cell subpopulations were highly positively correlated with genes such as G2M checkpoint, E2F targets, MYC targets v1 and mTORC1 signalling. G2M checkpoint is an important cell cycle‐related gene that is associated with the process of mitosis and is often abnormally expressed in various cancers.^29^ In addition, Hao et al. also found that MYC‐related signalling is related to cell differentiation and cell cycle, and abnormal MYC signalling pathway can lead to the proliferation, spread and enhancement of tumour dryness of LUAD.^30^ As a result, tumour cells may enhance the activity of mitosis‐related pathways, leading to radiotolerance and abnormal division.

We observed that the expression levels of many cell cycle‐related genes in two subtypes increased with pseudotime changes, such as PABPC1, MT‐ATP8, ID1, CENPF, MKI67 and TOP2A. Poly A‐binding protein cytoplasmic 1 (PABPC1) is a class of RNA‐binding proteins involved in the translation, metabolism and regulation of RNA. Abnormal expression of PABPC1 may lead to changes in the function of cells and tissues, leading to the occurrence and progression of cancer.^31^ Li et al. found that PABPC1 can be regulated by tumour suppressor MKRN3 to induce ubiquitination in NSCLC, thus affecting the progression of cell division cycle and controlling the proliferation and invasion of tumour cells.^32^ Mitochondrially encoded ATP synthase 8 (MT‐ATP8) is an important protein enzyme involved in mitochondrial ATP synthesis and energy conversion, which has important biological significance for cell metabolism and growth. Mutations and abnormal expression of mitochondrially encoded ATP have been found in breast cancer and other cancers.^33^, ^34^ The inhibitor of DNA‐binding 1 (ID1) was found to activate the NF‐κB signalling pathway, increase the expression level of IL‐6 in tumour tissues, and also increase the drug resistance and sensitivity of cancer cells to stimuli.^35^ Centromere protein F (CENPF), which may co‐regulate the mitosis and proliferation of cancer cells with the cell cycle‐related protein CDK1, has been found to be overexpressed in adrenal cortical carcinoma, which may be associated with poor prognosis.^36^ Type II topoisomerases (TOP2) are topoisomerases that can catalyse the breaking and joining of DNA. They are involved in a variety of chromosome activities, including DNA replication, transcription and repair, and are of great significance for cell division and proliferation. It can also lead to tumour‐related mutations.^37^

We found that the proportion of TP53 in patients who had received radiotherapy was significantly higher than that in patients who had not received radiotherapy, and the expression level of TP53 in patients who had received radiotherapy was significantly lower than that in patients who had not received radiotherapy. These suggest that radiotolerance is related to TP53 mutation to some extent. TP53 is a widely studied tumour suppressor gene. As an important transcriptional regulator, TP53 regulates important life activities such as cell cycle, programmed death and DNA damage, and has been found to be mutated in most cancers.^38^ TP53 alteration is detected in approximately 23%–65% of NSCLC cases and can be observed in up to 50.8% of LUADs.^39^, ^40^ The majority of mutations in TP53 occur in a hotspot region, specifically in exons 5–8, which are responsible for DNA binding.^41^ There exists a strong and positive correlation between the presence of missense‐type TP53 mutations and unfavourable survival outcomes in colorectal cancer patients, implicating a potential gain‐of‐function contribution by mutant p53 in driving tumour progression.^42^ TP53 missense mutations account for most TP53 mutations in LUADs and can result in the accumulation of mutant p53 due to increased protein stability.^43^ The analysis of this study indicated the presence of a high frequency of missense mutant p53 that may be involved in the improvement of patients receiving chemotherapy drugs. NSCLC patients with concomitant TP53 mutation experience poorer survival rates and respond less effectively to chemotherapy and radiation.^44^ Whereas some LUADs characterized by a singular TP53 mutation display a high‐grade fetal lung‐like appearance, there are isolated instances of NSCLC cases showing typical adenocarcinoma features that solely present TP53 mutation as a genetic aberration.^45^, ^46^ In addition, Sun et al. found that TP53 can also be used as a potential target for the development of immune checkpoint inhibitors to help LUAD patients achieve better clinical benefits.^43^ Although TP53 is a tumour suppressor gene, the lower levels of TP53 expression observed in LUAD patients after radiotherapy do not directly indicate mutations. It is also possible that the reduced expression of TP53 in patients who received radiotherapy could be influenced by other factors such as treatment‐induced stress, epigenetic changes or selection for radioresistant cell populations. Thus, further investigation is required to determine whether these reduced expression levels are a result of TP53 mutations or other regulatory mechanisms affected by radiotherapy.

In this study, we constructed a single cell atlas of A549 treated with radiation based on scRNA‐seq technology, and identified the cell subpopulations related to radiotolerance in TCGA‐LUAD. Further study found that radiotolerance was related to TP53 mutation. However, there are still major limitations in this project. At present, all our analysis data are from the public database, and the sample number is limited. In future, we need to validate our findings in clinical and cellular experiments to further investigate the molecular mechanisms by which TP53 mutations affect radiotolerance. We also look forward to our study providing a reference for personalized treatment for LUAD patients.

## 5 CONCLUSION

Based on the scRNA‐seq technique, we identified HIST1H1D+ A549 cells and PIF1+ A549 cells as the cell subpopulations related to radiotolerance, and found that the expression level of cell cycle‐related genes between the two subpopulations was significantly higher. Further study found that radiotolerance was related to TP53 mutation. We expect our study to help reveal the molecular mechanism of LUAD radiotolerance and personalize treatment for LUAD patients in the clinic.

## AUTHOR CONTRIBUTIONS

**Feiyun Chang:** Conceptualization (lead); investigation (lead); project administration (lead); supervision (lead); writing – original draft (lead); writing – review and editing (lead). **Bozhou Xi:** Investigation (equal); resources (equal); software (equal); supervision (equal). **Xinchun Chai:** Investigation (equal); methodology (equal); resources (equal); software (equal); validation (equal); visualization (equal). **Xiuyan Wang:** Investigation (equal); methodology (equal); resources (equal); visualization (equal). **Manyuan Ma:** Investigation (equal); project administration (equal); software (equal); validation (equal); visualization (equal). **Yafeng Fan:** Conceptualization (equal); data curation (equal); validation (equal); visualization (equal); writing – original draft (equal).

## FUNDING INFORMATION

The authors declare that they received no funding.

## CONFLICT OF INTEREST STATEMENT

The authors declare that they have no conflicts of interest regarding this manuscript.

## Supporting information


Data S1.

Figure S1.

## Data Availability

The public dataset used in this study is available in GSE211617 https://www.ncbi.nlm.nih.gov/geo/query/acc.cgi?acc=GSE211617.
