# Supplementary material for: Molecular mechanism of radiation tolerance in lung adenocarcinoma cells using single‐cell RNA sequencing
Source: J Cell Mol Med. 2024 May 17;28(10):e18378. doi: 10.1111/jcmm.18378 (PMC11101670; doi:10.1111/jcmm.18378)
Supplement: Supplementary file 1 — Data S1. [file JCMM-28-e18378-s001.docx]

**Description of supplementary figure legends**

**Figure S1** **Sample information for scRNA-seq data from the GSE211617 dataset.**

A: UMAP of the cells in each sample; B: Intracellular UMI number distribution of each sample; C: Distribution of the number of genes detected in the cells of each sample; D: Distribution of the proportion of intracellular mitochondrial genes in each sample
