# Supplementary figures and images for: Molecular mechanism of radiation tolerance in lung adenocarcinoma cells using single‐cell RNA sequencing
Source: J Cell Mol Med. 2024 May 17;28(10):e18378. doi: 10.1111/jcmm.18378 (PMC11101670; doi:10.1111/jcmm.18378)

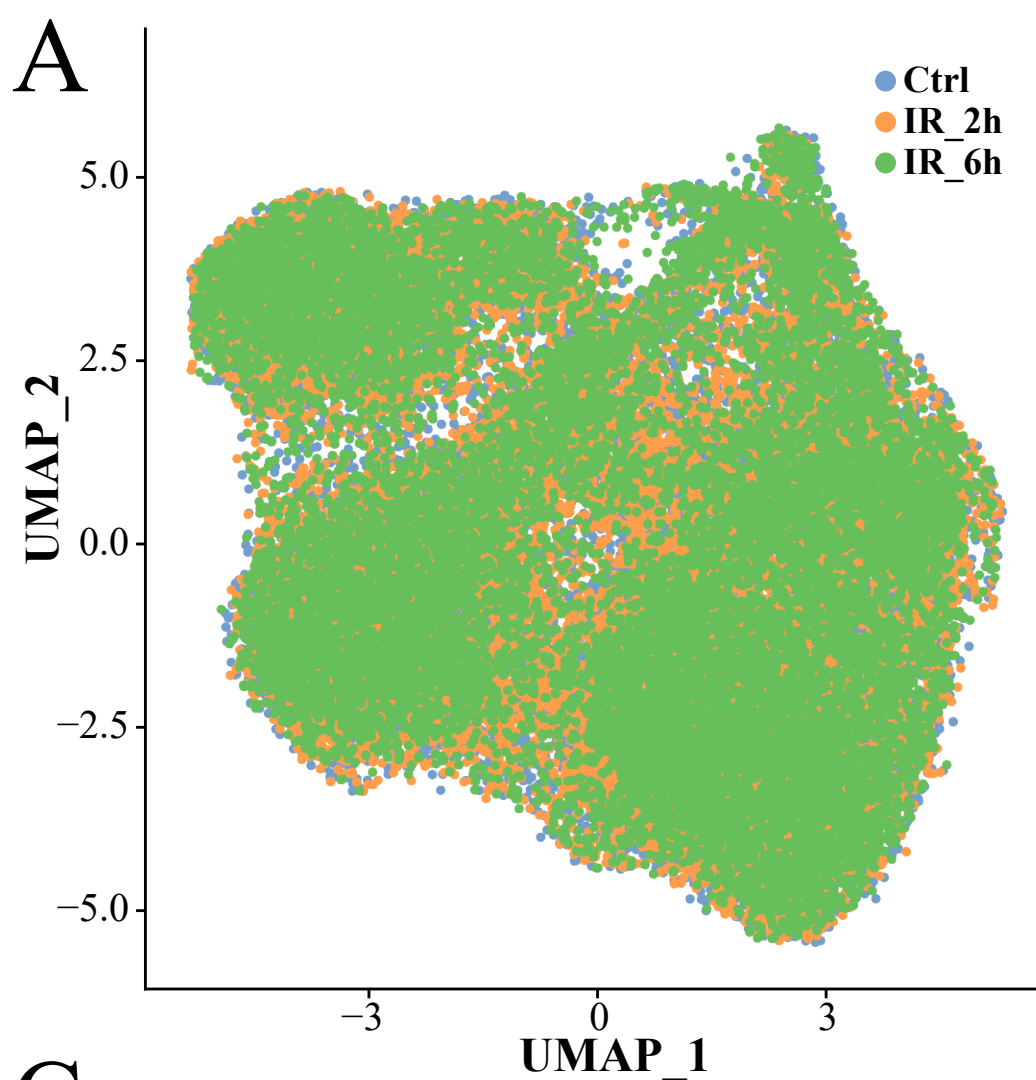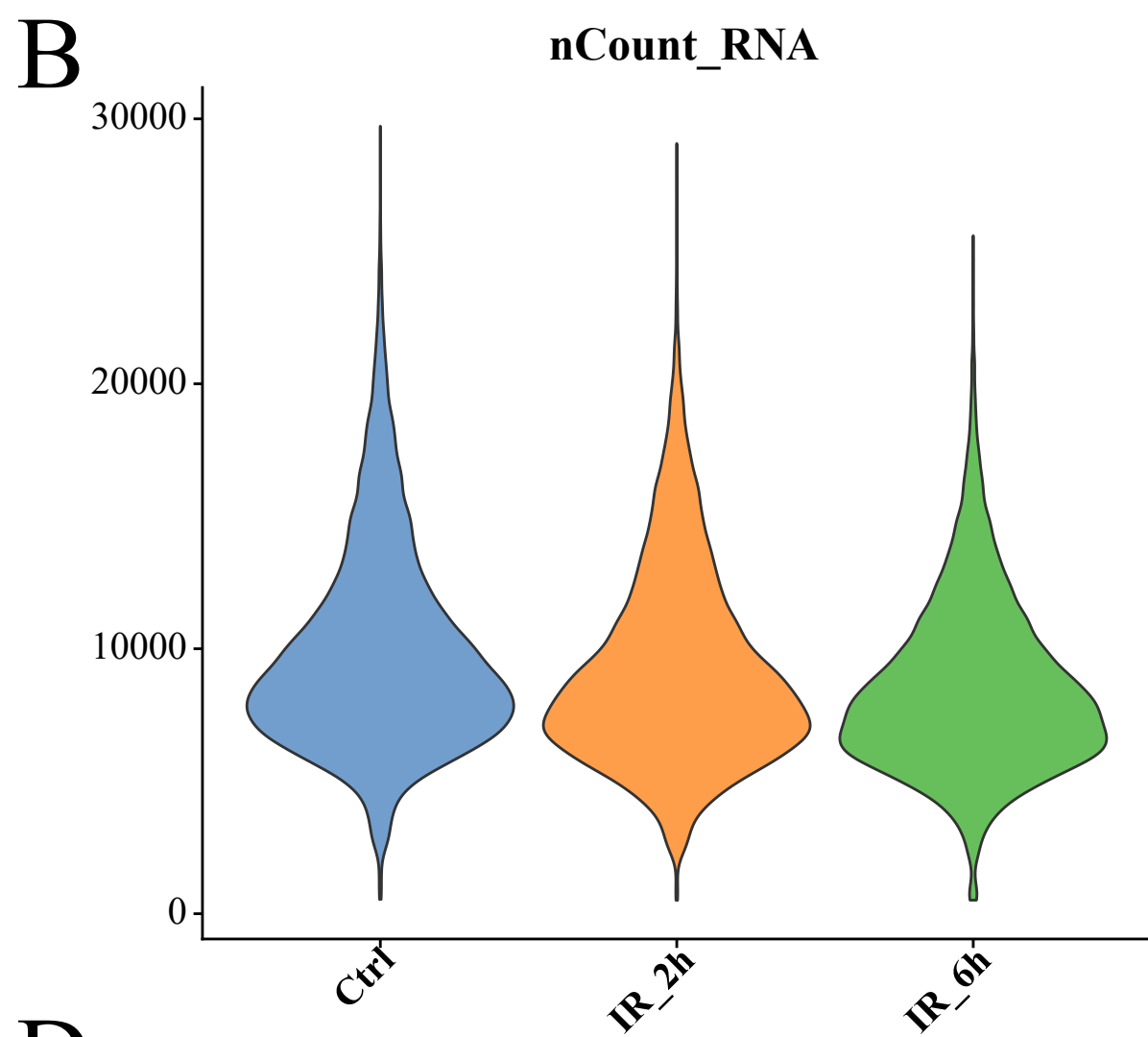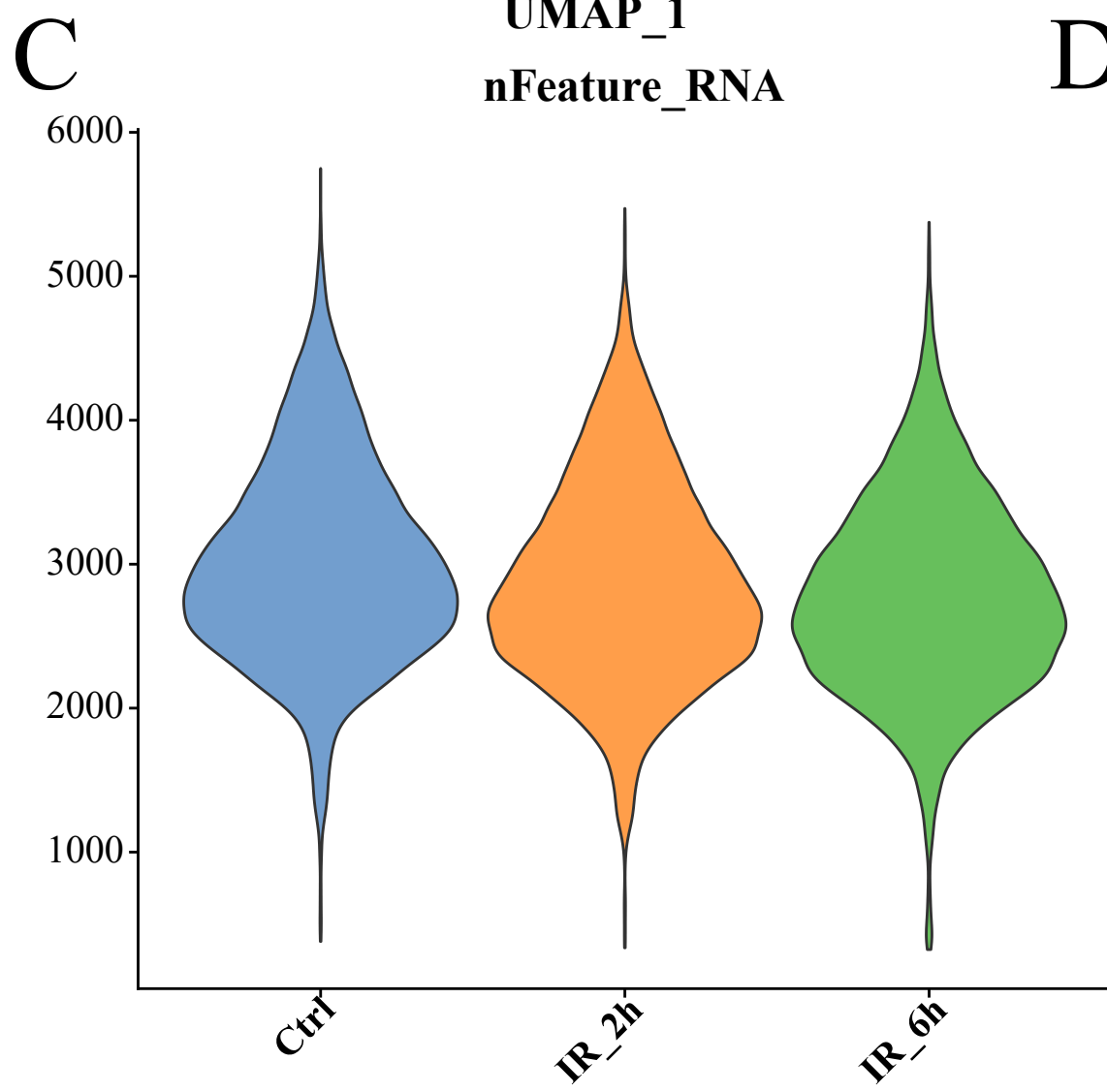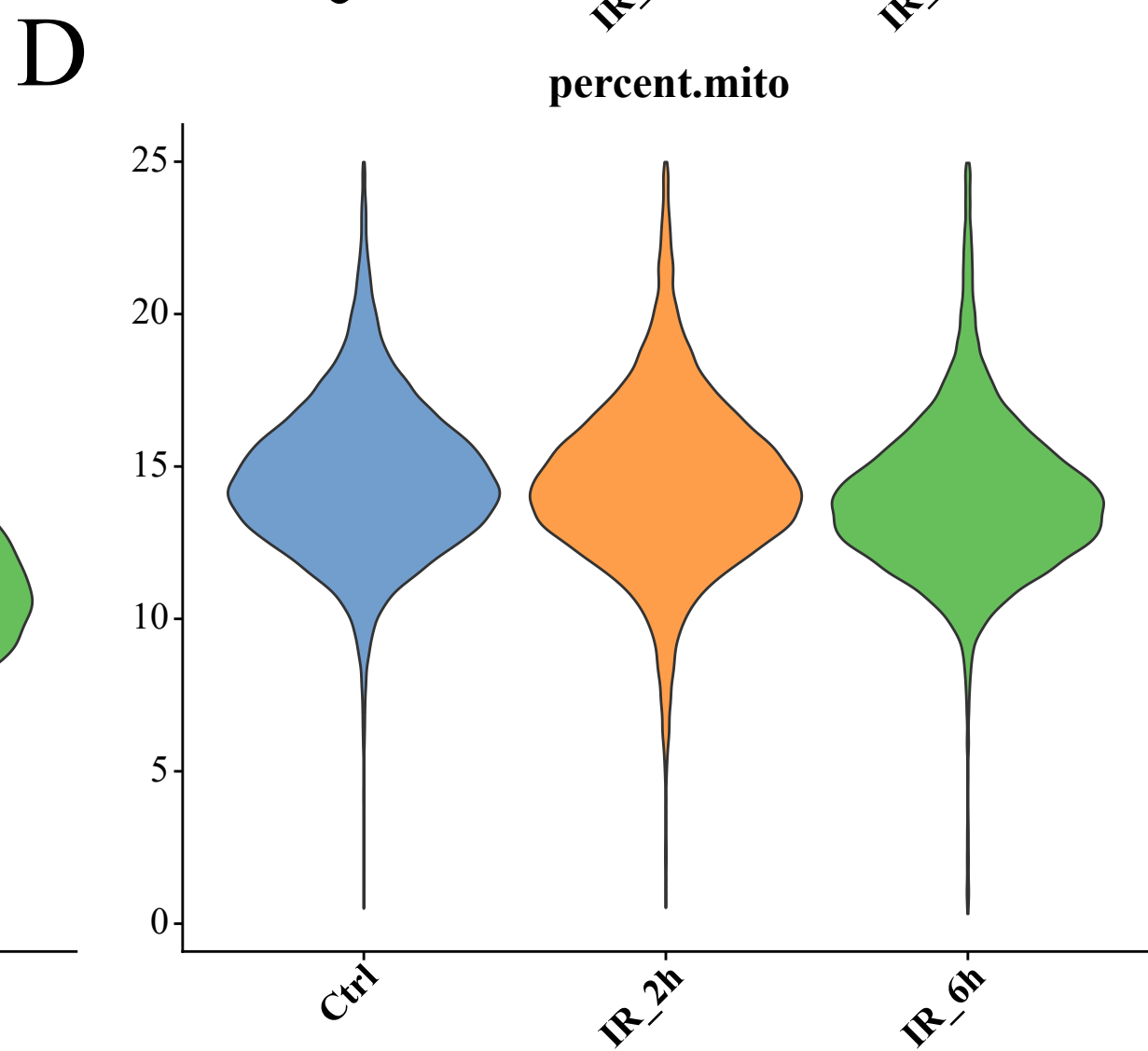

Supplement: Supplementary file 2 — Figure S1. [file JCMM-28-e18378-s002.pdf]
